# Politics and Research: a desirable and essential dialogue

**Published:** 2014-12-19

**Authors:** Maurizio Bifulco

**Affiliations:** President of Faculty of Pharmacy and Medicine, University of Salerno

An exhausting process of bureaucratization, an ever-changing stream of rules dictated by fluctuating succession of parliamentary majorities and new governments, an irregular system of evaluation and recruitment of new human resources: this is the photography, raw but real, of the current situation concerning University and Research in Italy. The alarm was recently launched in an open letter addressed to the Minister of Education, University and Research, signed by forty-eight senior members of the Accademia Nazionale dei Lincei, the oldest scientific academy in the world and the highest Italian cultural institution (1). A shared complaint, a careful reflection on the need to stem the Italian political power which, while intervening to prevent malfunctions and to accelerate the efficiency of research and training, limits its organizational autonomy enshrined in the Italian Constitution. An unequivocal answer to those who propose, once again, a reorganization more “political” than “scientific” for research: the unification of the Italian research centers, now twenty, under strict government control would put a limit on the freedom of research and teaching. The reasons, in an era of “spending review”, would be purely economic: cutting of the administrative agencies would result in a saving for the benefit of the State, although, according to an initial assessment, seems totally derisory, just 1 per thousand of the expenditure for public bodies (2). An unworkable political control, aimed at channeling through strict standards the reality of research, which by its nature requires a flexibility protecting its heterogeneous character. A betrayal, more or less conscious, of the task of government institutions, that is, to encourage the specificities of each subject area, to enhance the diversity of skills, to ensure self-government and self-discipline on the basis of the principle of responsibility of researchers, to encourage the exchange of knowledge, free from constraints, and the creation of networks of collaboration and coordination; to ensure, ultimately, the possibility of development, innovation and competitiveness for our country and a viable future for the new generations of researchers.

To exacerbate the already dramatic framework, indeed, the status of Italians doctoral students and post-docs who, according to a just published survey, at the end of their training would have very little chance to work in a more or less stable manner in the world of research and career prospects virtually nil (3). You can not and you should not, however, throw in the towel. Despite the very limited investments and increasing and unbridgeable misunderstandings between the institutional and scientific world, the numbers of research in Italy bode well. As reported the “Consolidator Grant Scheme 2013”, our country is at the second place in terms of European and non-European research projects funded by the European Research Council (4), and our researchers are the first in the world by number of articles published and citations pro-capite (5). Record numbers, which confirm, without doubts, the value of our research and our researchers, which is primarily social and cultural. To preserve this value and make it grow, it would be desirable openness to dialogue with public institutions, to look for a respectful separation of responsibilities and powers, political and technical, which, paradoxically, is now the only way to get a unity of purpose.
